# T. indotineae: A New Emergent Fungal Pathogen Driven by Global Travel

**DOI:** 10.1111/myc.70170

**Published:** 2026-03-29

**Authors:** Bryan Ortiz, Florent Morio, Kateryn Aguilar, Franklin García, Gustavo Fontecha, Alicia Moreno‐Sabater

**Affiliations:** ^1^ Instituto de Investigaciones en Microbiología, Facultad de Ciencias Universidad Nacional Autónoma de Honduras Tegucigalpa Honduras; ^2^ Nantes Université, CHU Nantes, Cibles et Médicaments Des Infections et de L'immunité, IICiMed, UR 1155 Nantes France; ^3^ Sorbonne Université, Centre D'immunologie et Des Maladies Infectieuses, (CIMI‐PARIS), Inserm U1135 Paris France; ^4^ Service de Parasitologie‐Mycologie, Hôpital Saint‐Antoine, AP‐HP Paris France

**Keywords:** dermatophytosis, migrants, *T. Mentagrophytes* ITS genotype VIII, terbinafine resistance, travel, *trichophyton indotineae*

## Abstract

**Background:**

The global spread of *Trichophyton indotineae*, a multidrug‐resistant dermatophyte, remains insufficiently understood due to limited data on migration‐associated dissemination and emerging local transmission. This study reviewed available evidence and integrated international migration statistics to assess the worldwide distribution of this pathogen and the influence of human mobility on its expansion.

**Methods:**

A systematic search in Scopus and PubMed (September 9, 2025) included epidemiological studies, case reports, and letters. Extracted variables comprised country of diagnosis, probable country of infection, and reported transmission scenarios. Migration data from the International Organization for Migration were incorporated to explore links between population movements and fungal spread, including indirect or undocumented routes.

**Results:**

Of the 224 publications identified, 89 met the inclusion criteria, reporting a total of 1215 cases outside India across 36 countries. Europe accounted for 545 cases, followed by North America with 90 cases and Oceania with 87 cases, while South America and Africa reported only sporadic detections. Fifty studies described imported infections, whereas 11 documented autochthonous transmission. Integrated analysis revealed three primary dissemination corridors: intra‐Asian, Asia–Europe, and Asia–America.

**Conclusions:**

*Trichophyton indotineae* is now globally disseminated and strongly shaped by travel and migration. Coordinated international action, standardized diagnostics, harmonized treatment guidelines, and integrated surveillance are urgently required to mitigate its expanding public health impact worldwide.

## 1 Introduction

Globalization has interconnected the world and facilitated trade, migration, and international transportation, driving economic, social, and cultural development. Yet this increasing interdependence also accelerates the spread of microorganisms, with significant implications for public health, economies, and the environment [1, 2, 3]. The recent SARS‐CoV‐2 pandemic clearly illustrated how quickly pathogens can spread across continents [4].

Among fungal infections associated with migration, the most prominent example is the spread of *Candida auris* (now *Candidozyma auris*), becoming a major public health concern due to its multidrug resistance and ability to cause outbreaks in healthcare settings [5]. Other fungal species have emerged in recent years, including *Trichophyton indotineae*, *T. mentagrophytes* genotype VII, and *T. benhamiae*, and their expansion is supposed to be associated with human movements and pet trade [6, 7, 8]. These emerging pathogens have given rise to novel clinical entities, posing increasing challenges for physicians [9].

*T. indotineae* has become a significant global public health concern due to its high transmissibility and alarming resistance to terbinafine, the first‐line treatment [10]. First described in 2020 (formerly classified as *T. mentagrophytes* ITS genotype VIII) [11], it affects individuals across all age groups, with clinical manifestations predominantly involving skin lesions, most commonly *tinea cruris* and *tinea corporis*. *T. indotineae* spreads readily within communities through direct human‐to‐human transmission and has occasionally been linked to clonal outbreaks [12]. Recent reports also suggest possible sexual transmission, similar to that reported for *T. mentagrophytes* genotype VII [13, 14].

The clinical relevance of this species clearly lies in its frequent resistance to terbinafine, a key factor in the outbreak of extensive and recalcitrant dermatophytosis reported in India in 2016 [15, 16]. *T. indotineae* can display acquired resistance to terbinafine, with reported rates reaching up to 75% in India [17, 18]. The leading hypothesis for the rise in terbinafine‐resistant *T. indotineae* links it to widespread misuse of over‐the‐counter creams containing corticosteroids, antibiotics, and antifungals. This inappropriate use likely exerts selective pressure that promotes the emergence of resistant strains [16, 19]. Currently, itraconazole is the recommended treatment of recalcitrant infections caused by for terbinafine‐resistant *T. indotineae* because griseofulvin and fluconazole show low efficacy [12, 20]. However, relapse is one of the most concerning and challenging aspects in the management of infections caused by *T. indotineae* as it can occur in up to 47% of cases [12].

A growing body of research has documented the rapid global spread of this dermatophyte and underscores the urgent need for public health interventions to prevent a recurrence of the crisis observed in India and to curb the further spread of terbinafine resistance [6, 7, 21]. However, the effective implementation of such action plans requires a more comprehensive understanding of the migration patterns associated with the dissemination of *T. indotineae*, as well as the identification of countries that are beginning to report endemic transmission. Such analyses are inherently complex as they require accurate identification of the infection source for each patient and depend heavily on the number and quality of studies conducted in each country, which may introduce bias. Moreover, because reporting of this emerging infection is not mandatory and no dedicated online surveillance systems exist, scientific publications remain the primary source of information. Considering these limitations and the need to understand the population dynamics involved in *T. indotineae* spread, we conducted a review of the existing literature and international reports concerning population movements to assess its current global distribution and the impact of human travel on its geographic expansion.

## 2 Materials and Methods

### 2.1 Mapping the Distribution of *T. indotineae*

To assess the global distribution and estimate the number of *T. indotineae* cases by country, a systematic literature review using the Scopus database (https://www.Scopus.com) and Pubmed (https://www.ncbi.nlm.nih.gov) on September 9, 2025, was conducted. The search terms included *Trichophyton indotineae*, *T. indotineae*, and *Trichophyton mentagrophytes* ITS genotype VIII, applied to titles, abstracts, and keywords. Studies were included based on the following criteria: epidemiological reports, case reports, and letters to the editor. No language restrictions were applied. The titles and corresponding DOIs were exported as a text file for further analysis. Study selection was conducted in two stages: (I) initial screening of titles, abstracts, and keywords and (II) full‐text review—both performed independently by two researchers. Relevant data were extracted into a structured matrix to facilitate systematic comparison and synthesis of findings.

### 2.2 Impact of Human Travel on the Geographical Expansion of *T. indotineae*

To estimate the expansion of *T. indotineae* beyond India, we extracted from each included report both the country where the diagnosis was made and the country where the infection was presumed to have occurred. For the purpose of this analysis, the presumed country of origin was defined as the patient's reported place of origin. However, in some cases, this may not have accurately reflected the actual location where the infection was acquired. These data were analysed to identify population flows potentially involved in the international spread of *T. indotineae*. Additionally, data from the International Organization for Migration (IOM) [https://worldmigrationreport.iom.int/msite/wmr‐2024‐interactive/?lang] were used to assess whether international migration patterns could be associated with the observed geographical expansion of the fungus. For each country in which *T. indotineae* infection was presumed to have originated, we obtained migration statistics indicating the top five destination countries, defined by the highest outbound migration flows, along with the respective migrant counts in 2020. Finally, unreported or indirect migratory routes declared in the https://weblog.iom.int/worlds‐congested‐human‐migration‐routes‐5‐maps were also recorded to also analysed their impact in the *T. indotineae* expansion.

## 3 Results

### 3.1 Estimated Geographic Distribution of *T. indotineae* in 2025

**FIGURE 1:**
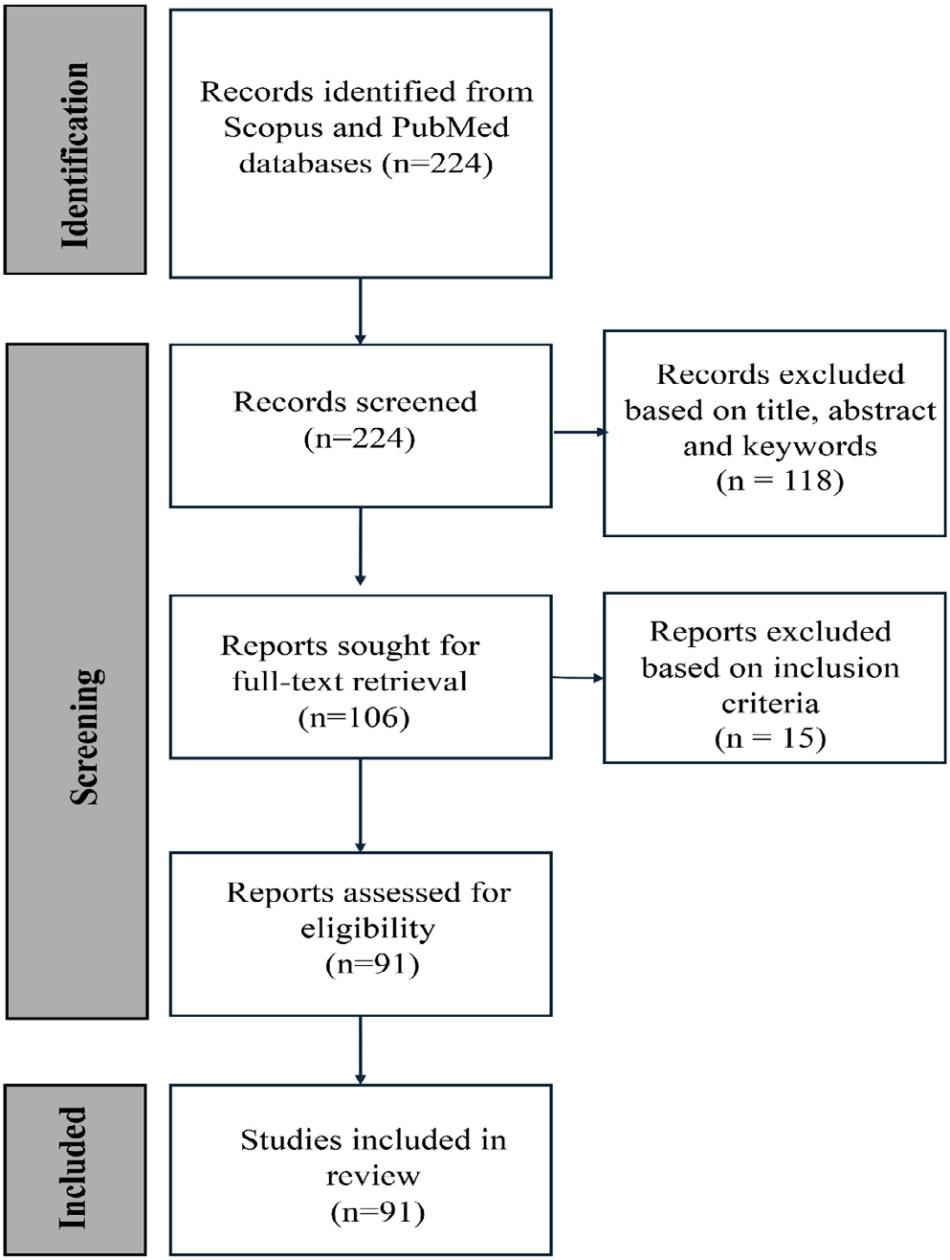
Flow chart illustrating the study selection process, including the number of records identified in Scopus and PubMed, screened, excluded, and finally included in the review.

**FIGURE 2:**
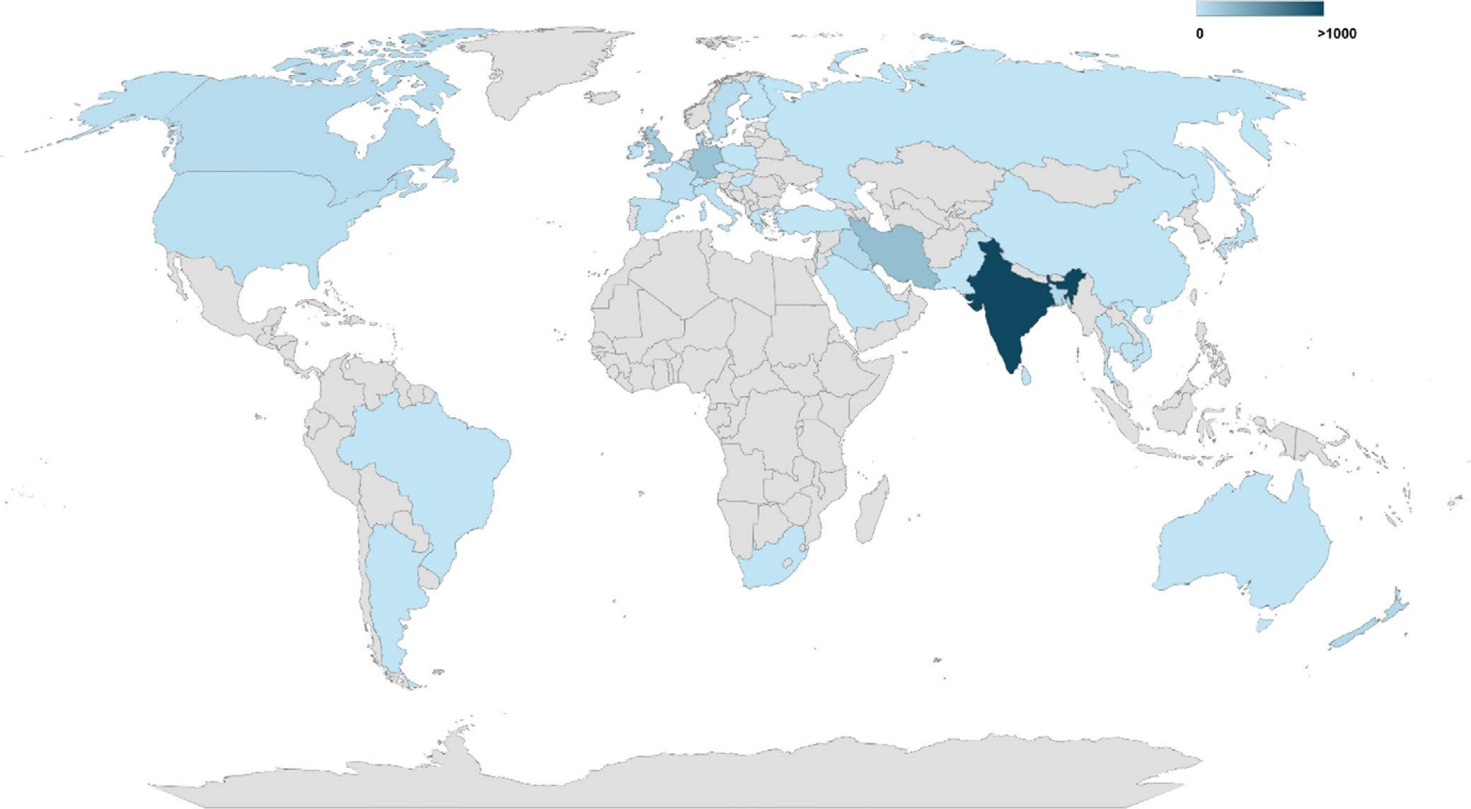
Estimated geographical distribution of *T. indotineae* in 2025. Heatmap representing the number of reported cases per country. Colour intensity reflects the number of reported *T. indotineae* cases in the literature.

The initial search retrieved 224 articles. Based on the title and abstract content, 108 articles were excluded. Full‐text analysis of the remaining 106 articles allowed us to retain 89 articles that met the inclusion criteria (Figure 1). Thirty‐six countries, with a total of 1215 confirmed cases, were documented outside of India (Figure 2). In Asia—excluding India—489 cases have been identified across 15 countries, accounting for approximately 40.2% of all travel‐associated infections. The highest numbers were reported in Iran (*n* = 239) [22, 23, 24, 25, 26, 27, 28], followed by Bangladesh (*n* = 76) [29] and Iraq (*n* = 70) [30, 31]. An increasing number of cases in China [32, 33] and Singapore [34] is also a cause for concern. Additional imported cases have been reported in Israel, Sri Lanka, Thailand, Malaysia, Cambodia, the United Arab Emirates, and Kuwait [7, 35, 36, 37, 38, 39, 40] (Supporting Information 1).

Although Southeast Asia is considered the primary focus of transmission of *T. indotineae*, with India recognized as an endemic country accounting for approximately 75% of dermatophytosis cases, Europe currently emerges as the second‐most affected continent. This is largely due to the higher number of epidemiological reports available from Europe compared with Asia, despite the well‐documented endemicity of *T. indotineae* in India [17, 18].

To date, Europe has reported a total of 545 confirmed cases of *T. indotineae* infection, with Germany (*n* = 219) and the United Kingdom (*n* = 158) accounting for most cases [41, 42, 43, 44, 45, 46]. Additional cases have been documented in Sweden (*n* = 51) [47], France (*n* = 29) [48, 49, 50, 51, 52, 53], Italy (*n* = 22) [54, 55, 56, 57, 58, 59], Spain (*n* = 20) [60, 61, 62, 63], and Switzerland (*n* = 15) [64, 65, 66]. A smaller number of cases were reported in Denmark (*n* = 10) [67, 68], Greece (*n* = 9) [69], The Czech Republic (*n* = 7) [70], and Russia (*n* = 2) [71]. Single cases were reported in Hungary [72], Ireland [73], and Belgium [74].

Nevertheless, further epidemiological studies in Southeast Asia and across the broader Asian continent are urgently needed to accurately reflect the true burden of *T. indotineae*. Although available evidence clearly supports its endemic status in this region, the limited number of epidemiological investigations suggests substantial underreporting, particularly in India, where the current data likely underestimate the real magnitude of *T. indotineae* circulation in Asia.

In North America, 90 cases have been reported to date, including 35 in the United States [13, 75, 76, 77, 78, 79] and 55 in Canada [80, 81]. In South America, only three cases have been documented, two in Brazil [82, 83] and one in Argentina [84]. Similarly, a single case has been reported for the whole African continent, described in South Africa [85]. In Oceania, numerous cases of *T. indotineae* have been reported in New Zealand (*n* = 85) [86], and to a lesser extent in Australia (*n* = 2) [87]. Analysis of reported cases by continent revealed that Europe has the highest number of *T. indotineae* cases after Asia. To date, Africa and South America remain the only continents with the lowest cases reported. All reports included in this study, broken down by country and organized by continent, are presented in Supporting Information 1.

### 3.2 Imported vs. Autochthonous Cases of *T. indotineae*

Different epidemiological scenarios were associated with the origin or transmission of *T. indotineae*. Among the 89 studies included in this review, 50 were exclusively associated with imported infections, while 11 studies described only autochthonous transmission. In nine studies, both imported and autochthonous cases were reported, and in the remaining 18 studies, no information was provided regarding the mode of transmission, and one study suggested zoonotic transmission [88] (Supporting Information 2).

Interestingly, local transmission was reported in countries not considered endemic for *T. indotineae* (i.e., outside India, Bangladesh, and Pakistan), such as Singapore, Thailand, Japan, Sri Lanka, China, Kuwait, Turkey, and Vietnam, suggesting that imported cases likely facilitated the establishment of autochthonous transmission [33, 36, 37, 39, 89, 90, 91, 92]. Regarding transmission routes, interhuman transmission was the main reported route, usually in a family context. Sexual contact appears to be an emerging mode of transmission [13, 50], and one report suggests the possibility of infection following contact with an animal [88].

### 3.3 Tracing Patient Movements to Understand *T. indotineae* Expansion

Given that most of the studies were associated with imported infections and to aim for a better understanding of the expansion of *T. indotineae*, patient movements were traced. To this end, the country where the infection was presumed to have been acquired and the country where the diagnosis was made were associated and analysed.

Countries where the infection was reportedly acquired were first examined. For 189 of the 1215 cases included in the analysis, it was possible to retrieve this information. These 189 patients had acquired the infection in 24 different countries (Table 1).

**TABLE 1:** Travel‐associated cases of *Trichophyton indotineae* infections according to the country where the infection is expected to have been contracted.

	Estimated Country/Region of infection Source	Number of cases associated	Country of case identification	References
Asian Countries	India	United Kingdom, Germany, Italy, France, Spain, Israel, Switzerland, China, Japan, Canada, United States, Singapore, Hungary, Czech Republic, Vietnam, and South Africa	79	[11, 21, 35, 41, 42, 44, 45, 48, 52, 53, 55, 57, 58, 61, 62, 64, 65, 66, 70, 72, 80, 85, 93, 94, 95, 96]
	Bangladesh	United Kingdom, United States, Ireland, Italy, France, Switzerland, Germany, and Spain,	63	[42, 45, 48, 49, 53, 54, 57, 58, 59, 60, 61, 62, 63, 65, 73, 76, 78, 97]
	Pakistan	United Kingdom, Italy, Germany, and Spain,	12	[42, 59, 62]
	Sri Lanka	United Kingdom, Australia, Italy, and France	6	[45, 48, 54, 57, 58, 87]
	Nepal	United Kingdom, Japan, Italy, and Spain	5	[11, 45, 56, 62, 98]
	Afghanistan	Australia and Switzerland	2	[66, 87]
	Iraq	Germany	2	[42]
	Iran	United Kingdom and Greece	2	[45, 69]
	Vietnam	Japan and Czech Republic	2	[70, 99]
	Bahrain	Germany	1	[42]
	Lebanon	Sweden	1	[47]
	Philippines	Japan	1	[100]
	Myanmar	France	1	[53]
	Singapore	Malaysia	1	[7]
	Syria	Greece	1	[69]
	United Arab Emirates	United Kingdom	1	[45]
	Turkey	United States	1	[75]
African Countries	Egypt	Italy	1	[59]
	Morocco	Spain	1	[60]
	Libya	Germany	1	[42]
American Countries	Peru	Italy	2	[54]
	Mexico	Argentina	1	[84]
European Countries	Spain	Sweden	1	[47]
	Portugal	France	1	[50]

The majority of patients (*n* = 181) were from Asia, representing 17 different countries. Most notably, cases were reported from India, Bangladesh, and Pakistan (*n* = 154), indicating a significant movement of people from these regions. Additional cases came from Sri Lanka, Nepal, Afghanistan, Iraq, Iran, and Vietnam (*n* = 19), and to a lesser extent from Bahrain, Lebanon, the Philippines, Myanmar, Singapore, Syria, the United Arab Emirates, and Turkey. Interestingly, a small number of patients reported acquiring the infection in African countries, such as Egypt, Morocco, and Libya (*n* = 3); in the Americas (*n* = 3); and in European countries such as Spain and Portugal (*n* = 2).

**FIGURE 3:**
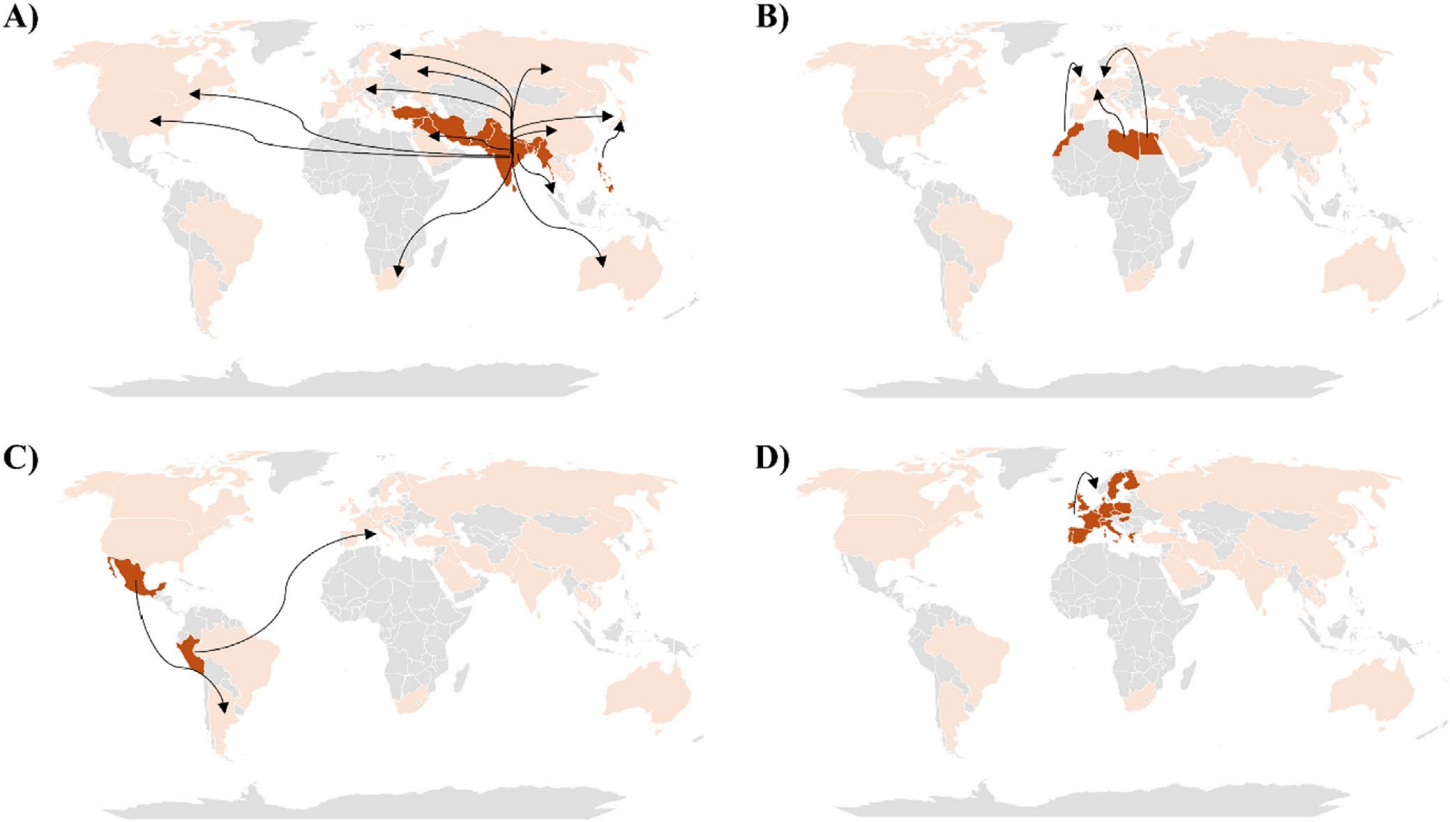
International population flows contributing to the global spread of *T. indotineae* based on the literature review. (A) Population movements originating from Asian countries—mainly India, Bangladesh, and Pakistan—suggest the existence of three major transmission corridors: Intra‐Asian, Asiatic–European, and Asiatic–American. (B–D) Population movements from North Africa to Europe, from South America, and within Europe indicate additional potential corridors originating outside Asia. Arrows represent the routes between the presumed source of infection and the country where the disease was diagnosed.

Countries where infections were diagnosed in these 189 patients were then analysed (Figure 3). Infections in patients presumed to be infected or to be of Asian origin were reported in 22 different countries, including six located in Asia (Israel, China, Japan, Singapore, Vietnam, and Malaysia), suggesting population movements that may contribute to the spread of *T. indotineae* within Asia (Table 1) (Figure 3A). Notably, 11 of the 22 were European countries (United Kingdom, Germany, Italy, France, Spain, Switzerland, Ireland, Czech Republic, Hungary, Greece, and Sweden), indicating a significant population flow between Asia and Europe that could facilitate the expansion of the fungus. To a lesser extent, cases of *T. indotineae* infection in patients of Asian origin were also reported in two North American countries (United States and Canada), one Oceanian country (Australia), and one African country (South Africa). These data suggest that the spread of *T. indotineae* mainly follows three population routes: an intra‐Asian corridor, an Asiatic–European corridor, and an Asiatic–American corridor.

*T. indotineae* in patients presumed to be infected or originating from African, American, or European countries appears to contribute to its spread to a lesser extent, involving 6 out of 22 countries (Table 1) (Figure 3B–D). Patients from African countries (*n* = 3) were diagnosed in Europe (notably Italy, Spain, and Germany). Patients from American countries (*n* = 3) were diagnosed in Argentina and Italy, while two patients from European countries were diagnosed in other European countries such as Sweden and France, suggesting an intra‐European transmission and migratory flows of the fungus. The analysis of these patient movements suggests other potential routes, such as the Mediterranean, American, or intra‐European corridors but the number of reported cases remains too limited to confirm their existence.

### 3.4 Understanding *T. indotineae* Expansion Through International Migratory Flows Analysis

**FIGURE 4:**
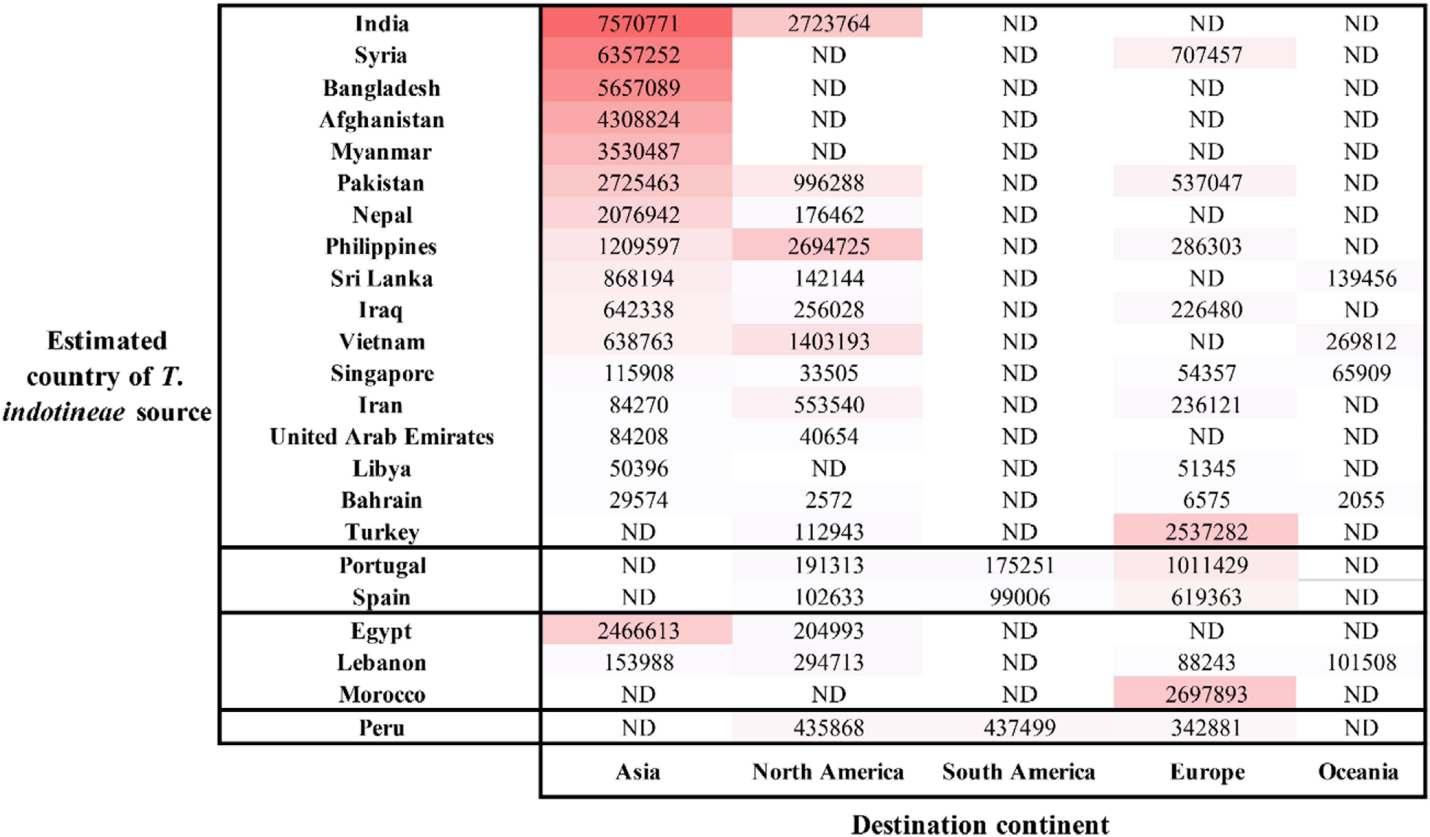
International migratory flows potentially associated with the spread of *T. indotineae*. Data shown in the tables were obtained from the *World Migration Report 2024*. International Organization for Migration (IOM), Geneva. McAuliffe M. and L.A. Oucho (eds.), 2024. International migratory flows from Mexico were significantly higher than those from other countries and were predominantly directed toward the United States (*n* = 10,853,105). This predominance limited the visualization of other migration patterns in the heat map; consequently, these flows were not represented. ND: No data were reported.

The impact of international migratory flows on the geographical expansion of *T. indotineae* observed in this study was also explored, based on data from the World Migration Report 2024 [93]. Population movements for each country in which *T. indotineae* infection was presumed to have originated were recorded (Figure 4).

Asian countries where *T. indotineae* infections are thought to have originated showed the highest levels of population movement compared to regions from Europe, the Americas, and Africa. Notably, migration from Asian countries such as India, Syria, and Bangladesh predominantly occurred within Asia, confirming the significant intra‐Asian mobility. Populations from the Philippines, India, Iran, and Pakistan also migrated to American countries, mainly to North America. Migration from South America to Europe was also observed. Europe received substantial migration flows from Asian countries such as Turkey, Syria, and Pakistan, as well as from Morocco, while notable intra‐European movements were also recorded. Finally, population flows to Oceania were comparatively low. Comparison of international migration flows described in the *World Migration Report* with patient movement patterns observed in this study reveals striking similarities between global migratory routes and the *T. indotineae* transmission corridors identified here.

From this analysis, two other main findings could be drawn. First, a high level of migration between India and the United States contrasted with the low number of *T. indotineae* cases reported in the United States compared to the higher number of cases reported in Europe, suggesting that the number of *T. indotineae* cases is underreported in Unites states. Second, the relatively low migration flow from Asian countries where *T. indotineae* has been documented to Europe contrasts with the high number of cases detected in Europe. These observations suggest that the spread of *T. indotineae* could follow unreported or indirect migratory routes.

## 4 Discussion

Estimating the true global incidence of *T. indotineae* infections remains difficult due to the scarcity of comprehensive epidemiological studies and the absence of prospective surveillance programs. Based on available reports, this review highlights that the global spread of *T. indotineae* appears to occur on three distinct levels. The first includes countries where endemicity has been demonstrated by epidemiological studies, such as Iran and Bangladesh [22, 23, 29, 94]. The neighbouring country Pakistan may also be endemic, although the absence of epidemiological data prevents confirmation [6, 18, 95]. The second level comprises countries where *T. indotineae* cases have been reported, notably across Asia, Europe, North America, and Oceania. The third level involves countries where infections were detected in patients originating from areas without officially reported cases, including Afghanistan, Bahrain, the Philippines, Lebanon, Myanmar, Nepal, Syria, Morocco, Egypt, Libya, Pakistan, Peru, and Portugal [43, 45, 47, 53, 54, 59, 60, 69, 87, 96]. Altogether, these findings indicate that *T. indotineae* has achieved a global distribution, at different levels, affecting all five continents.

Effective public health policies require understanding not only the geographic distribution of *T. indotineae* but also the population movements driving its spread. To date, the role of migratory dynamics in the dissemination of this fungus has not been systematically investigated as no official reporting system exists to capture such data. However, analysis of case reports, combined with official statistics on international migration, has enabled the identification in this review of three main corridors associated with patient movements and the expansion of *T. indotineae*. The intra‐Asian corridor of *T. indotineae* can be associated with the South Asian subregion, which includes Afghanistan, Bangladesh, Bhutan, India, Iran, Maldives, Nepal, Pakistan, and Sri Lanka [7, 97]. This area experiences significant internal and international migration [98, 99]. In 2020, an estimated 14 million international migrants resided within the subregion, 11 millions of whom originated from neighbouring countries, reflecting intense intraregional mobility. Humanitarian crises, such as the displacement of Rohingya refugees in Bangladesh and Afghan refugees in Pakistan and Iran [93, 99, 100], have likely facilitated the dissemination of *T. indotineae* to countries bordering India [93, 101].

Established international migration routes linking South Asia to North America and Europe have also allow to define a Asian–Noth America corridor and an Asian–Europe corridor of *T. indotineae*, which could explain the transcontinental spread of this fungus [102]. Interestingly, the expansion of *T. indotineae* appears particularly pronounced in Europe, which accounts for the majority of reported infections outside Asia. Although this may reflect Europe's advanced diagnostic systems, the explanation is weakened by the lower number of cases reported in North America, where healthcare standards are similarly high. International migration could also contribute while many European cases originate from infections acquired in India or Bangladesh; migration data suggest indirect population flows as Europe is not among the main destinations for migrants from these countries. The analysis of migration routes from countries presumed to be the origin of patient infections in this study indicates that Europe primarily receives incoming populations via Turkey and Morocco. Since these countries are not currently considered endemic for *T. indotineae*, they may serve as transit zones for infected individuals travelling along these countries. Turkey and Marocco have been described as transit zones for infected individuals travelling along the Eastern Mediterranean and Central Mediterranean routes—pathways historically used by people fleeing conflict or poverty in the Middle East and Africa (https://weblog.iom.int/worlds‐congested‐human‐migration‐routes‐5‐maps). These observations raise the hypothesis that irregular migration could contribute to the introduction and spread of *T. indotineae* in Europe.

The presented analysis also showed that population flows from South Asian subregion to Africa and Latin American countries appear to be less significant. Indeed, an epidemiological study conducted in Mexico failed to identify any *T. indotineae* cases despite the country's high migratory activity [103]. Certainly, in this region, there is a predominance of intra‐American migration—from South America and the Northern Triangle (El Salvador, Guatemala, and Honduras)—with relatively limited migration from Asia, which may restrict the spread of *T. indotineae* to this region. Finally, it must be noted that African countries are not among the top five destinations for Asian migrants, which could further explain the limited presence of this fungus on the continent. However, definitive conclusions remain difficult to draw due to the scarcity of epidemiological studies conducted to date.

In summary, *T. indotineae* has expanded globally from Asia to Europe, the Americas, and Oceania, mainly through travel and migration. Underreporting persists due to limited surveillance. A coordinated international response is urgently needed, with standardized diagnostics, treatment guidelines, and integrated monitoring. Including *T. indotineae* in existing fungal disease frameworks will be crucial to limit its global health impact.

## Author Contributions

**Bryan Ortiz:** conceptualization, methodology, data curation, formal analysis, writing – original draft, supervision. **Kateryn Aguilar:** data curation, formal analysis, writing – review and editing. **Florent Morio:** methodology, resources, writing – review and editing. **Franklin García:** data curation, validation, writing – review and editing. **Alicia Moreno‐Sabater:** conceptualization, methodology, data curation, formal analysis, writing – original draft, supervision.

## Funding

This study did not receive any specific grant from funding agencies in the public, commercial, or not‐for‐profit sectors.

## Conflicts of Interest

The authors declare no conflicts of interest.

## Supporting information


**Supporting Information: 1** Global reports included in this study, organized by country and continent.
**Supporting Information:** 2. Global reports of Trichophyton indotineae infections indicating country of diagnosis, suspected country of origin, migration or travel history, and reference DOI.

## Data Availability

The data that supports the findings of this study are available in the Suppporting Information of this article.
